# Adenoid cystic carcinoma of the palate: case report and review of literature

**DOI:** 10.11604/pamj.2016.24.106.8596

**Published:** 2016-05-31

**Authors:** Uday Shankar Yaga, Nishanth Gollamudi, Ashwini Kumar Mengji, Radhika Besta, Prashanth Panta, Bhanu Prakash, Edunuri Rajashekar

**Affiliations:** 1 Department of Oral Medicine and Radiology, MNR Dental College and Hospital, Sangareddy, Telangana, India

**Keywords:** Cribriform variant, palate, perineural invasion, salivary gland

## Abstract

Adenoid Cystic Carcinoma (ACC) is a rare tumor constitutes for less than 1% of head and neck malignancies and 10% of all salivary gland tumors. Palate is the most common site to be involved in the oral cavity followed by parotid gland and submandibular gland. They are usually asymptomatic, slow growing, characteristically shows infiltrative growth and perineural invasion. This paper reports a case of Adenoid Cystic Carcinoma in a 35 year old female man reported with a swelling on the left side of palate involving the hard and soft palate since 8 months which was diagnosed histopathologically and review of literature of the peculiar clinical, and histopathological features.

## Introduction

Adenoid cystic carcinoma is a malignant neoplasm that may affect either the major or minor salivary glands of the oral cavity [1, 2]. It was first described by three Frenchmen (Robin, Lorain, and Laboulbene) in two articles published in 1853 and 1854. It was they who described the cylindrical appearance of this tumor. Billroth, in1859, first described ACC under the name “cylindroma”, for its cribriform appearance formed by tumor cells with cylindrical pseudolumina or pseudospaces and described that ACC had a “great tendency to recur [3].” It accounts for about 5% to 10% of all salivary gland neoplasms, representing 2% to 4% of malignant occurrences of the head and neck area. Approximately 31% of lesions affect minor salivary glands, particularly the palate, though they can also be observed in the submandibular and parotid glands [4]. Although it presents a widespread age distribution, peak incidence occurs predominantly among women, between the 5^th^ and 6th decades of life [5]. Typical clinical findings include slow growth, local recurrence, perineural invasion and distant metastasis [6]. Three histological subtypes of ACC are known: cribriform, tubular and solid. They may occur either separately or together in the same tumor, and the solid subtype is the most aggressive [7]. A unique feature of ACC is the propensity for perineural invasion, even with early-stage tumors. Tumor is graded according to Szanto et al. [8] cribriform or tubular (grade I), less than 30% solid (grade II), or greater than 30% solid (grade III). It is currently recognized that ACC remains an extremely difficult disease to treat. It was described by Conley and Dingman as “one of the most biologically destructive and unpredictable tumors of the head and neck [9]”. We report a case of adenoid cystic carcinoma of palate and a brief literature review on its clinical, histopathological and therapeutic aspects.

## Patient and observation

**Figure 1:**
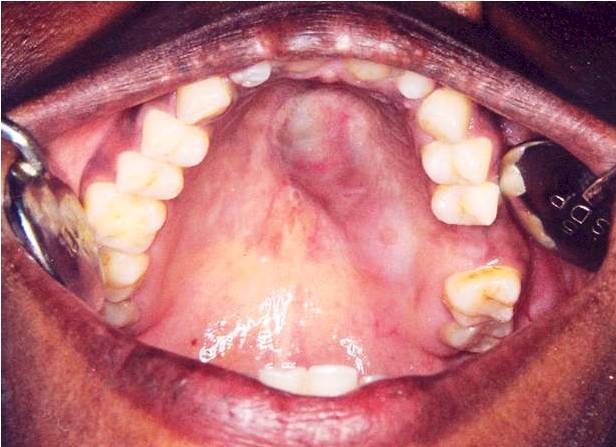
Intra-oral picture showing swelling extending from right central incisor to left second molar

**Figure 2:**
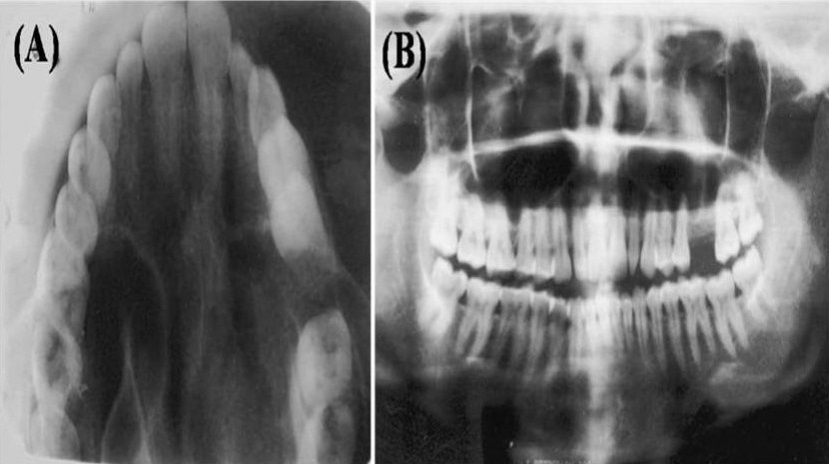
(A) maxillary occlusal radiograph; (B) panoramic radiograph

**Figure 3:**
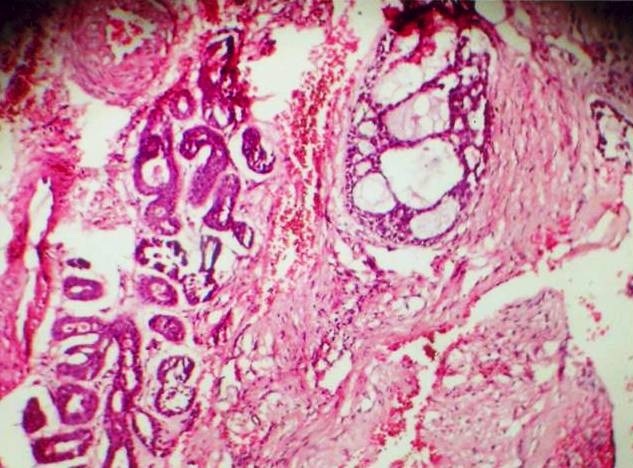
H & E view showing shows isomorphic basoloid tumor cells in cribriform pattern with eosinophilic material

A 35 year old female patient came to the department of oral medicine, diagnosis and radiology with a chief complaint of swelling in upper back jaw region since 8 months. She presents with history of difficulty in mastication and deglutition. The swelling was small initially and gradually increased to the present size. She also gives history of extraction irt 26 due to decay 5 years back. Intra oral examination revealed a single well defined swelling extending antero-posteriorly from upper right central incisor to left second molar which was crossing the midline, filling the palatal vault and involving both hard and soft palate (Figure 1). There was no mobility of the involved teeth. No surface discharge was present. On palapation, the inspectory findings were confirmed. The swelling was non-tender, non-fluctuant, hard in consistency and immobile. Routine blood investigations were found to be normal. A provisional diagnosis of minor salivary gland tumor was established. No bony changes were observed in occlusal and panoramic radiographs (Figure 2). An incisional biopsy was performed under local anesthesia and was subjected to histopathological examination. Microscopically it shows isomorphic basoloid tumor cells in cribriform pattern with eosinophilic material (10x) (Figure 3). A diagnosis of Adenoid Cystic Carcinoma (cribriform pattern) was established.

## Discussion

The adenoid cystic carcinoma constitutes approximately 29.6% of minor salivary gland tumors. Often palate is the most commonly involved site, tongue being the second most common; other sites are floor of mouth, and lip [10]. ACC occurs most often in minor salivary glands and the submandibular gland, and less frequently in the sublingual and parotid glands. Other rare locations include the aerodigestive tract, minor salivary glands, lachrymal glands and adnexal skin glands [7]. Rarely, it may also present as primary intraosseous tumors of the maxilla and mandible. ACC is thought to arise from the mucous-secreting glands. It arises specifically from the intercalated ducts, and electron microscopy shows that it arises from cells that can differentiate into epithelial and myoepithelial cells. These mucus-secreting tumors are confined to structures derived from the foregut (that is, the parotid, submandibular, and sublingual glands, and the mucus glands throughout the upper respiratory tract) [7]. Most frequent clinical feature of adenoid cystic carcinoma affecting major salivary gland is reported to be the presence of tumor-usually 2-4 cm at its greatest diameter and intraoral adenoid cystic carcinoma seldom larger than 3 cm at its greatest diameter [11, 12]. The lesion is uncapsulated and infiltrative; invasion of underlying bone is common. Incidence of cervical metastasis is low. Distant metastasis occurs through blood stream to lung and bones. Direct extension of lesion of the base of skull has been reported as a cause of death [12, 13].

Histopathologically it presents three patterns, cribriform, tubular and solid; the most common variant is the cribriform pattern, in which the epithelial cells are arranged in multiple cylindrical spaces, having a pseudo cystic appearance, and many of these pseudo cysts contain a hyaline material. The tubular type is made up of ducts that can be formed by one or two layers of cells similar to the myoepithelial cells. The solid variant is composed of solid epithelial islands with central areas of necrosis; the cells are small, basophilic and hyperchromatic with a densely granulated nucleus and scarce mitotic figures [14]. The solid type has a poor prognosis contrary to the cribriform type which has a better prognosis. Surgical excision with wide margins is the treatment of choice and, when it metastasizes to the lymph nodes, post surgical radiotherapy is recommended. The most important prognostic factors include primary lesion size (T), anatomical localization, presence or absence of metastasis (M) at diagnosis time, invasion of the facial nerve and the histopathology grade (G) [14]. Possible treatments of ACC include four different modalities: surgical therapy, radiotherapy, chemotherapy and combined therapy (surgery and radiotherapy, radiotherapy and chemotherapy), being the latter in most cases, the treatment of choice [15, 16]. Only surgical removal or radiotherapy in isolation may fail to eliminate the possibility of recidivation in surgical margins, as well as the occurrence of metastasis in cervical lymph nodes, lungs, bones and brain. In addition, ACC presents a strong neurotropism, with a tendency to invade nerves adjacent to the lesion [10]. Salivary gland neoplasms form a diverse group of tumors, with different histological characteristics and clinical behavior patterns. This marked variation in histological grading and clinical classification means that the evaluation of these tumors requires extensive knowledge of anatomy and physiology, as well as expertise in pathology [17]. Long-term follow up of patients with salivary gland neoplasms is mandatory because of the frequently indolent but relentlessly infiltrative behaviour associated with late locoregional recurrence and distant metastasis of adenoid cystic carcinoma.

## Conclusion

Salivary gland tumors should be considered in the differential diagnosis of aggressive lesions in maxilla and mandible and especially when the aggressive lesion is involving palate, adenoid cystic carcinoma involving minor salivary gland tumors should be considered in differential diagnosis. The primary treatment objective in adenoid cystic carcinoma patients is local control, normal functionality and distant metastasis prevention. For this purpose, early detection of the lesion is a requirement, in order to enable a more favorable prognosis and better quality of life.
